# 6-Acetoxy­methyl-3-[(2-hydr­oxy-3-methoxy­benzyl­idene)amino]-3,4,5,6-tetra­hydro-2*H*-pyran-2,4,5-triyl triacetate

**DOI:** 10.1107/S1600536810011475

**Published:** 2010-03-31

**Authors:** Yan Fei Wang, Shu-Hua Zhang, Zhen Feng Chen, Hong Liang

**Affiliations:** a College of Chemistry and Chemical Engineering, Central South University, Changsha 410083, People’s Republic of China; b School of Chemistry and Chemical Engineering, Guangxi Normal University, Guilin 541004, People’s Republic of China; c College of Chemistry and Bioengineering, Guilin University of Technology, Guilin 541004, People’s Republic of China

## Abstract

The title compound, C_22_H_27_NO_11_, was synthesized by the reaction of 4,5-diacet­oxy-6-acetoxy­methyl-3-amino­tetra­hydro­pyran-2-yl acetate and 2-hydr­oxy-3-methoxy­benzalde­hyde in ethanol. The mol­ecule contains two six-membered rings, one of which is in a chair conformation, and an intra­molecular O—H⋯N hydrogen bond is present.

## Related literature

For a Schiff base complex, see: Zhang *et al.* (2003 ▶). For macrocyclic Schiff base compounds, see: Frischmann *et al.* (2008 ▶); Jiang *et al.* (2010 ▶). For 5,5′-dimeth­oxy-2,2′-[4,5-dimethyl-*o*-phenyl­enebis(nitrilo­methyl­idyne)]diphenol, which shows similar hydrogen-bonding to the title compound, see: Kargar *et al.* (2010 ▶).
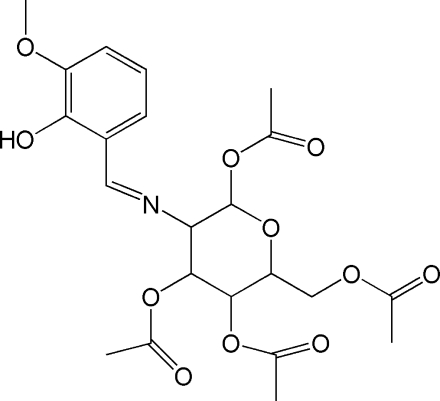


## Experimental

### 

#### Crystal data

C_22_H_27_NO_11_
*M* *_r_* = 481.45
Orthorhombic, 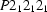

*a* = 10.806 (3) Å
*b* = 11.151 (3) Å
*c* = 20.243 (5) Å
*V* = 2439.2 (11) Å^3^
*Z* = 4
Mo *K*α radiation
μ = 0.11 mm^−1^
*T* = 296 K
0.32 × 0.28 × 0.22 mm

#### Data collection

Bruker SMART CCD area-detector diffractometer
12313 measured reflections
2460 independent reflections
1506 reflections with *I* > 2σ(*I*)
*R*_int_ = 0.061

#### Refinement

*R*[*F* ^2^ > 2σ(*F* ^2^)] = 0.046
*wR*(*F* ^2^) = 0.126
*S* = 1.03
2460 reflections
314 parameters
H-atom parameters constrained
Δρ_max_ = 0.17 e Å^−3^
Δρ_min_ = −0.14 e Å^−3^

### 

Data collection: *SMART* (Bruker 2004 ▶); cell refinement: *SAINT* (Bruker, 2004 ▶); data reduction: *SAINT*; program(s) used to solve structure: *SHELXS97* (Sheldrick, 2008 ▶); program(s) used to refine structure: *SHELXL97* (Sheldrick, 2008 ▶); molecular graphics: *SHELXTL* (Sheldrick, 2008 ▶); software used to prepare material for publication: *SHELXL97*.

## Supplementary Material

Crystal structure: contains datablocks global, I. DOI: 10.1107/S1600536810011475/si2250sup1.cif

Structure factors: contains datablocks I. DOI: 10.1107/S1600536810011475/si2250Isup2.hkl

Additional supplementary materials:  crystallographic information; 3D view; checkCIF report

## Figures and Tables

**Table 1:** Hydrogen-bond geometry (Å, °)

*D*—H⋯*A*	*D*—H	H⋯*A*	*D*⋯*A*	*D*—H⋯*A*
O1—H1⋯N1	0.82	1.90	2.625 (4)	147

**Fig. 1.**
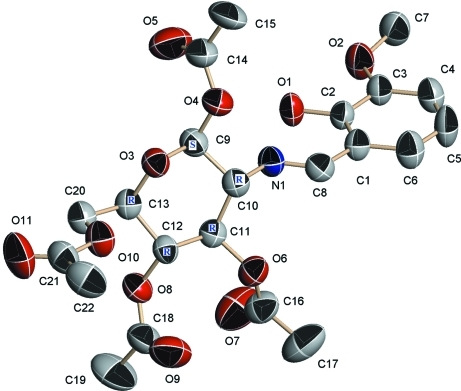
The molecular structure of (I), showing 30 % probability displacement ellipsoids. H-atoms were omitted.

## supplementary crystallographic information

### Comment

Schiff base compounds (Zhang, *et al.* 2003; Frischmann, *et al.* 2008; Jiang, *et al.* 2010) have aroused increasing interest because of their antiviral, anticancer and antibacterial activities. Herein, we report the synthesis and crystal structure of a new schiff base compound, (I), prepared by the reaction of Acetic acid 4,5-diacetoxy-6-acetoxymethyl-3-amino]-tetrahydro-pyran-2-yl ester and 2-hydroxy-3-methoxy-benzaldehyde.

The molecular structure of (I) reveals the 2-hydroxy-3-methoxy-benzaldehyde configuration with one acetic acid 4,5-diacetoxy-6-acetoxymethyl-3-amino]-tetrahydro-pyran-2-yl ester molecule on N1-position (Fig. 1). The dihedral angle between the benzene ring of 2-hydroxy-3-methoxy-benzylidene group and the plane of C9, C10, C12, C13 is 56.78 (3) °. The other dihedral angle between the four acetic acid groups and the plane of C9, C10, C12, C13 are in the range of 57.0–111.7°. There is an intramolecular O–H···N hygrogen bond between the phenol and imido-group(Table 1). The distance of N1···H1 is substantially shorter than the van der Waals distance of 2.75 Å for the N and H distance. The hydrogen bond between the phenol and imido-group are similar to those found in the crystal structure of 5,5'-Dimethoxy-2,2'-[4,5-dimethyl-*o*- phenylenebis(nitrilomethylidyne)]diphenol (Kargar, *et al.* 2010). In the molecule, the C9 has S* configuration, while the C10, C11, C12, C13 are in R* configuration which form a R* configuration molecule.

### Experimental

The compound Acetic acid 4,5-diacetoxy-6-acetoxymethyl- 3-amino]-tetrahydro-pyran-2-yl ester (0.182 g, 0.5 mmol) was dissolved in ethanol (10 ml). To this solution, 2-hydroxy-3-methoxy-benzaldehyde (0.076 g, 1 mmol) was added and the mixture was stirred and refluxed at 333 K for 3 h. After cooling to room temperature and filtration, the filtrate was left to stand at room temperature. Colourless block crystals suitable for X-ray diffraction were obtained in a yield of 53 %. Analysis found (%): C 54.64, H 5.69, N 2.94; C_22_H_27_NO_11_ requires (%) : C 54.88, H 5.65, N 2.91.

### Refinement

All H atoms were positioned geometrically and were refined as riding, with (C—H 0.93–0.98 Å, O—H 0.82 Å with *U*_iso_(H) = 1.2 *U*_eq_(aromatic C) and *U*_iso_(H) = 1.5*U*_eq_(other C or O).

In the absence of significant anomalous dispersion effects, Friedel pairs were averaged.

### Crystal data

**Table:**

C_22_H_27_NO_11_	*F*(000) = 1016
*M**_r_* = 481.45	*D*_x_ = 1.311 Mg m^−^^3^
Orthorhombic, *P*2_1_2_1_2_1_	Mo *K*α radiation, λ = 0.71073 Å
Hall symbol: P 2ac 2ab	Cell parameters from 1384 reflections
*a* = 10.806 (3) Å	θ = 2.6–18.6°
*b* = 11.151 (3) Å	µ = 0.11 mm^−^^1^
*c* = 20.243 (5) Å	*T* = 296 K
*V* = 2439.2 (11) Å^3^	Block, colourless
*Z* = 4	0.32 × 0.28 × 0.22 mm

### Data collection

**Table:**

Bruker SMART CCD area-detector diffractometer	1506 reflections with *I* > 2σ(*I*)
Radiation source: fine-focus sealed tube	*R*_int_ = 0.061
graphite	θ_max_ = 25.1°, θ_min_ = 2.0°
phi and ω scans	*h* = −12→12
12313 measured reflections	*k* = −12→13
2460 independent reflections	*l* = −24→24

### Refinement

**Table:**

Refinement on *F*^2^	Secondary atom site location: difference Fourier map
Least-squares matrix: full	Hydrogen site location: inferred from neighbouring sites
*R*[*F*^2^ > 2σ(*F*^2^)] = 0.046	H-atom parameters constrained
*wR*(*F*^2^) = 0.126	*w* = 1/[σ^2^(*F*_o_^2^) + (0.0572*P*)^2^ + 0.177*P*] where *P* = (*F*_o_^2^ + 2*F*_c_^2^)/3
*S* = 1.03	(Δ/σ)_max_ < 0.001
2460 reflections	Δρ_max_ = 0.17 e Å^−^^3^
314 parameters	Δρ_min_ = −0.14 e Å^−^^3^
0 restraints	Extinction correction: *SHELXL97* (Sheldrick, 2008), Fc^*^=kFc[1+0.001xFc^2^λ^3^/sin(2θ)]^-1/4^
Primary atom site location: structure-invariant direct methods	Extinction coefficient: 0.0074 (13)

### Special details

**Table:**

Geometry. All esds (except the esd in the dihedral angle between two l.s. planes) are estimated using the full covariance matrix. The cell esds are taken into account individually in the estimation of esds in distances, angles and torsion angles; correlations between esds in cell parameters are only used when they are defined by crystal symmetry. An approximate (isotropic) treatment of cell esds is used for estimating esds involving l.s. planes.
Refinement. Refinement of F^2^ against ALL reflections. The weighted R-factor wR and goodness of fit S are based on F^2^, conventional R-factors R are based on F, with F set to zero for negative F^2^. The threshold expression of F^2^ > 2sigma(F^2^) is used only for calculating R-factors(gt) etc. and is not relevant to the choice of reflections for refinement. R-factors based on F^2^ are statistically about twice as large as those based on F, and R- factors based on ALL data will be even larger.

### Fractional atomic coordinates and isotropic or equivalent isotropic displacement parameters (Å^2^)

**Table:**

	*x*	*y*	*z*	*U*_iso_*/*U*_eq_	
N1	0.4441 (3)	0.0108 (3)	−0.01092 (15)	0.0566 (9)	
O1	0.2954 (3)	−0.1155 (2)	−0.08619 (14)	0.0650 (8)	
H1	0.3485	−0.1041	−0.0580	0.098*	
O2	0.1136 (3)	−0.1188 (3)	−0.17050 (16)	0.0796 (10)	
O3	0.5742 (3)	−0.0540 (2)	0.15220 (13)	0.0587 (8)	
O4	0.3813 (3)	0.0041 (3)	0.12705 (14)	0.0679 (8)	
O5	0.2923 (4)	−0.1724 (4)	0.1455 (2)	0.1216 (16)	
O6	0.7049 (3)	0.0885 (2)	−0.01748 (13)	0.0653 (8)	
O7	0.7871 (5)	−0.0297 (4)	−0.09480 (18)	0.1221 (16)	
O8	0.8528 (3)	−0.1002 (3)	0.05512 (15)	0.0654 (8)	
O9	0.9690 (4)	0.0649 (4)	0.0508 (2)	0.1069 (14)	
O10	0.8042 (3)	−0.0141 (3)	0.21533 (14)	0.0709 (9)	
O11	0.9237 (4)	−0.1041 (4)	0.2885 (2)	0.1134 (15)	
C1	0.3183 (4)	0.0980 (4)	−0.09509 (19)	0.0564 (11)	
C2	0.2611 (4)	−0.0094 (4)	−0.11268 (17)	0.0523 (10)	
C3	0.1641 (4)	−0.0085 (4)	−0.1582 (2)	0.0579 (11)	
C4	0.1275 (5)	0.0970 (4)	−0.1868 (2)	0.0686 (13)	
H4	0.0620	0.0974	−0.2166	0.082*	
C5	0.1877 (6)	0.2026 (4)	−0.1716 (2)	0.0830 (17)	
H5	0.1651	0.2735	−0.1925	0.100*	
C6	0.2796 (5)	0.2030 (4)	−0.1261 (2)	0.0763 (15)	
H6	0.3178	0.2752	−0.1153	0.092*	
C7	0.0039 (5)	−0.1240 (5)	−0.2101 (2)	0.0794 (15)	
H7A	−0.0601	−0.0770	−0.1898	0.119*	
H7B	−0.0230	−0.2057	−0.2139	0.119*	
H7C	0.0213	−0.0927	−0.2533	0.119*	
C8	0.4107 (4)	0.1028 (4)	−0.0435 (2)	0.0593 (11)	
H8	0.4473	0.1762	−0.0338	0.071*	
C9	0.4872 (4)	−0.0535 (4)	0.1006 (2)	0.0590 (11)	
H9	0.4679	−0.1351	0.0859	0.071*	
C10	0.5319 (4)	0.0245 (4)	0.0434 (2)	0.0545 (11)	
H10	0.5334	0.1086	0.0575	0.065*	
C11	0.6604 (4)	−0.0123 (4)	0.02084 (19)	0.0537 (10)	
H11	0.6556	−0.0841	−0.0070	0.064*	
C12	0.7471 (4)	−0.0339 (3)	0.07825 (18)	0.0524 (10)	
H12	0.7737	0.0427	0.0972	0.063*	
C13	0.6865 (4)	−0.1114 (4)	0.13127 (19)	0.0562 (11)	
H13	0.6663	−0.1898	0.1123	0.067*	
C14	0.2886 (5)	−0.0675 (6)	0.1493 (3)	0.0823 (16)	
C15	0.1874 (5)	0.0050 (6)	0.1787 (3)	0.1032 (19)	
H15A	0.1311	−0.0468	0.2018	0.155*	
H15B	0.1438	0.0466	0.1443	0.155*	
H15C	0.2217	0.0622	0.2091	0.155*	
C16	0.7680 (6)	0.0677 (5)	−0.0739 (2)	0.0792 (15)	
C17	0.8086 (7)	0.1835 (6)	−0.1032 (3)	0.120 (2)	
H17A	0.8882	0.1734	−0.1234	0.180*	
H17B	0.8140	0.2432	−0.0692	0.180*	
H17C	0.7498	0.2086	−0.1359	0.180*	
C18	0.9585 (5)	−0.0393 (6)	0.0418 (3)	0.0821 (15)	
C19	1.0539 (6)	−0.1225 (6)	0.0159 (3)	0.124 (2)	
H19A	1.0194	−0.1692	−0.0194	0.187*	
H19B	1.0809	−0.1750	0.0507	0.187*	
H19C	1.1231	−0.0773	−0.0003	0.187*	
C20	0.7666 (5)	−0.1294 (4)	0.1910 (2)	0.0670 (13)	
H20A	0.7209	−0.1721	0.2249	0.080*	
H20B	0.8388	−0.1766	0.1794	0.080*	
C21	0.8859 (5)	−0.0145 (5)	0.2653 (2)	0.0731 (14)	
C22	0.9208 (6)	0.1090 (5)	0.2857 (3)	0.0973 (18)	
H22A	0.8510	0.1476	0.3059	0.146*	
H22B	0.9463	0.1540	0.2476	0.146*	
H22C	0.9878	0.1053	0.3168	0.146*	

### Atomic displacement parameters (Å^2^)

**Table:**

	*U*^11^	*U*^22^	*U*^33^	*U*^12^	*U*^13^	*U*^23^
N1	0.062 (2)	0.055 (2)	0.0534 (19)	0.0008 (19)	−0.0100 (17)	−0.0002 (18)
O1	0.069 (2)	0.0494 (16)	0.077 (2)	0.0002 (16)	−0.0246 (18)	0.0102 (14)
O2	0.083 (2)	0.061 (2)	0.095 (2)	−0.0107 (18)	−0.037 (2)	0.0081 (17)
O3	0.060 (2)	0.0645 (18)	0.0515 (17)	0.0054 (15)	−0.0011 (16)	0.0003 (13)
O4	0.0571 (19)	0.075 (2)	0.0714 (18)	0.0045 (19)	0.0075 (17)	0.0013 (17)
O5	0.099 (3)	0.096 (3)	0.170 (4)	−0.015 (3)	0.035 (3)	0.016 (3)
O6	0.074 (2)	0.0671 (18)	0.0549 (17)	−0.0070 (17)	−0.0021 (17)	0.0039 (15)
O7	0.175 (5)	0.109 (3)	0.083 (2)	−0.002 (3)	0.043 (3)	−0.015 (2)
O8	0.057 (2)	0.0658 (18)	0.0736 (19)	0.0114 (17)	0.0010 (16)	−0.0063 (16)
O9	0.073 (3)	0.095 (3)	0.153 (4)	−0.014 (2)	0.009 (3)	−0.006 (3)
O10	0.080 (2)	0.0673 (19)	0.0651 (17)	0.0067 (19)	−0.0188 (18)	−0.0055 (15)
O11	0.113 (4)	0.111 (3)	0.116 (3)	0.022 (3)	−0.055 (3)	0.003 (3)
C1	0.067 (3)	0.049 (2)	0.054 (2)	0.004 (2)	−0.008 (2)	0.000 (2)
C2	0.057 (3)	0.050 (2)	0.049 (2)	0.001 (2)	−0.005 (2)	0.0066 (19)
C3	0.062 (3)	0.052 (2)	0.059 (2)	0.002 (2)	−0.011 (2)	0.005 (2)
C4	0.072 (3)	0.064 (3)	0.070 (3)	0.009 (3)	−0.019 (3)	0.003 (2)
C5	0.107 (5)	0.051 (3)	0.091 (3)	0.006 (3)	−0.042 (4)	0.013 (2)
C6	0.094 (4)	0.047 (2)	0.088 (3)	−0.004 (3)	−0.030 (3)	−0.001 (2)
C7	0.073 (4)	0.079 (3)	0.085 (3)	−0.017 (3)	−0.026 (3)	0.014 (3)
C8	0.060 (3)	0.054 (2)	0.064 (3)	−0.002 (2)	−0.004 (2)	−0.005 (2)
C9	0.054 (3)	0.066 (3)	0.057 (3)	0.004 (2)	−0.002 (2)	−0.001 (2)
C10	0.056 (3)	0.051 (2)	0.057 (2)	−0.003 (2)	−0.007 (2)	−0.004 (2)
C11	0.059 (3)	0.047 (2)	0.055 (2)	−0.003 (2)	−0.001 (2)	−0.004 (2)
C12	0.054 (3)	0.050 (2)	0.053 (2)	0.007 (2)	−0.001 (2)	−0.0019 (18)
C13	0.062 (3)	0.053 (2)	0.054 (2)	0.002 (2)	−0.006 (2)	−0.002 (2)
C14	0.062 (4)	0.103 (4)	0.082 (3)	0.000 (3)	0.007 (3)	0.019 (3)
C15	0.051 (3)	0.151 (5)	0.108 (4)	0.009 (4)	0.012 (3)	0.021 (4)
C16	0.088 (4)	0.092 (4)	0.058 (3)	−0.012 (3)	0.000 (3)	0.003 (3)
C17	0.147 (7)	0.131 (5)	0.083 (4)	−0.049 (5)	0.010 (4)	0.017 (4)
C18	0.060 (4)	0.101 (4)	0.086 (4)	0.012 (3)	0.007 (3)	0.004 (3)
C19	0.083 (4)	0.158 (6)	0.132 (5)	0.035 (5)	0.023 (4)	−0.031 (5)
C20	0.072 (3)	0.063 (3)	0.066 (3)	0.006 (3)	−0.009 (3)	0.000 (2)
C21	0.067 (3)	0.088 (4)	0.064 (3)	0.006 (3)	−0.013 (3)	−0.006 (3)
C22	0.079 (4)	0.119 (5)	0.094 (4)	−0.002 (4)	−0.017 (3)	−0.028 (4)

### Geometric parameters (Å, °)

**Table:**

N1—C8	1.271 (5)	C7—H7B	0.9600
N1—C10	1.461 (5)	C7—H7C	0.9600
O1—C2	1.351 (4)	C8—H8	0.9300
O1—H1	0.8200	C9—C10	1.526 (6)
O2—C3	1.368 (5)	C9—H9	0.9800
O2—C7	1.433 (5)	C10—C11	1.519 (6)
O3—C9	1.406 (5)	C10—H10	0.9800
O3—C13	1.435 (5)	C11—C12	1.512 (5)
O4—C14	1.359 (6)	C11—H11	0.9800
O4—C9	1.417 (5)	C12—C13	1.525 (6)
O5—C14	1.172 (6)	C12—H12	0.9800
O6—C16	1.350 (6)	C13—C20	1.501 (6)
O6—C11	1.448 (5)	C13—H13	0.9800
O7—C16	1.184 (6)	C14—C15	1.485 (7)
O8—C18	1.356 (6)	C15—H15A	0.9600
O8—C12	1.438 (5)	C15—H15B	0.9600
O9—C18	1.182 (6)	C15—H15C	0.9600
O10—C21	1.343 (5)	C16—C17	1.486 (7)
O10—C20	1.435 (5)	C17—H17A	0.9600
O11—C21	1.176 (5)	C17—H17B	0.9600
C1—C6	1.393 (6)	C17—H17C	0.9600
C1—C2	1.394 (5)	C18—C19	1.483 (7)
C1—C8	1.446 (6)	C19—H19A	0.9600
C2—C3	1.395 (5)	C19—H19B	0.9600
C3—C4	1.370 (6)	C19—H19C	0.9600
C4—C5	1.380 (6)	C20—H20A	0.9700
C4—H4	0.9300	C20—H20B	0.9700
C5—C6	1.354 (6)	C21—C22	1.487 (7)
C5—H5	0.9300	C22—H22A	0.9600
C6—H6	0.9300	C22—H22B	0.9600
C7—H7A	0.9600	C22—H22C	0.9600
			
C8—N1—C10	119.4 (3)	O8—C12—C13	106.2 (3)
C2—O1—H1	109.5	C11—C12—C13	111.4 (3)
C3—O2—C7	117.9 (3)	O8—C12—H12	110.1
C9—O3—C13	110.4 (3)	C11—C12—H12	110.1
C14—O4—C9	117.0 (4)	C13—C12—H12	110.1
C16—O6—C11	119.2 (4)	O3—C13—C20	108.0 (3)
C18—O8—C12	118.5 (3)	O3—C13—C12	108.6 (3)
C21—O10—C20	116.2 (4)	C20—C13—C12	113.3 (4)
C6—C1—C2	118.3 (4)	O3—C13—H13	109.0
C6—C1—C8	120.2 (4)	C20—C13—H13	109.0
C2—C1—C8	121.5 (4)	C12—C13—H13	109.0
O1—C2—C1	122.0 (3)	O5—C14—O4	122.6 (6)
O1—C2—C3	118.3 (4)	O5—C14—C15	126.5 (6)
C1—C2—C3	119.7 (4)	O4—C14—C15	110.8 (5)
O2—C3—C4	125.4 (4)	C14—C15—H15A	109.5
O2—C3—C2	114.4 (3)	C14—C15—H15B	109.5
C4—C3—C2	120.2 (4)	H15A—C15—H15B	109.5
C3—C4—C5	120.1 (4)	C14—C15—H15C	109.5
C3—C4—H4	120.0	H15A—C15—H15C	109.5
C5—C4—H4	120.0	H15B—C15—H15C	109.5
C6—C5—C4	120.1 (4)	O7—C16—O6	123.2 (5)
C6—C5—H5	120.0	O7—C16—C17	127.0 (5)
C4—C5—H5	120.0	O6—C16—C17	109.7 (5)
C5—C6—C1	121.6 (4)	C16—C17—H17A	109.5
C5—C6—H6	119.2	C16—C17—H17B	109.5
C1—C6—H6	119.2	H17A—C17—H17B	109.5
O2—C7—H7A	109.5	C16—C17—H17C	109.5
O2—C7—H7B	109.5	H17A—C17—H17C	109.5
H7A—C7—H7B	109.5	H17B—C17—H17C	109.5
O2—C7—H7C	109.5	O9—C18—O8	122.9 (5)
H7A—C7—H7C	109.5	O9—C18—C19	127.1 (6)
H7B—C7—H7C	109.5	O8—C18—C19	110.0 (5)
N1—C8—C1	122.8 (4)	C18—C19—H19A	109.5
N1—C8—H8	118.6	C18—C19—H19B	109.5
C1—C8—H8	118.6	H19A—C19—H19B	109.5
O3—C9—O4	105.2 (3)	C18—C19—H19C	109.5
O3—C9—C10	110.7 (3)	H19A—C19—H19C	109.5
O4—C9—C10	106.5 (3)	H19B—C19—H19C	109.5
O3—C9—H9	111.4	O10—C20—C13	108.6 (3)
O4—C9—H9	111.4	O10—C20—H20A	110.0
C10—C9—H9	111.4	C13—C20—H20A	110.0
N1—C10—C11	109.8 (3)	O10—C20—H20B	110.0
N1—C10—C9	107.9 (3)	C13—C20—H20B	110.0
C11—C10—C9	111.4 (3)	H20A—C20—H20B	108.3
N1—C10—H10	109.3	O11—C21—O10	122.1 (5)
C11—C10—H10	109.3	O11—C21—C22	126.0 (5)
C9—C10—H10	109.3	O10—C21—C22	111.9 (5)
O6—C11—C12	109.3 (3)	C21—C22—H22A	109.5
O6—C11—C10	104.8 (3)	C21—C22—H22B	109.5
C12—C11—C10	112.2 (3)	H22A—C22—H22B	109.5
O6—C11—H11	110.1	C21—C22—H22C	109.5
C12—C11—H11	110.1	H22A—C22—H22C	109.5
C10—C11—H11	110.1	H22B—C22—H22C	109.5
O8—C12—C11	108.9 (3)		
			
C6—C1—C2—O1	178.2 (4)	C16—O6—C11—C12	100.4 (4)
C8—C1—C2—O1	−5.2 (6)	C16—O6—C11—C10	−139.2 (4)
C6—C1—C2—C3	−2.7 (6)	N1—C10—C11—O6	77.7 (4)
C8—C1—C2—C3	173.9 (4)	C9—C10—C11—O6	−162.9 (3)
C7—O2—C3—C4	−8.2 (7)	N1—C10—C11—C12	−163.8 (3)
C7—O2—C3—C2	172.3 (4)	C9—C10—C11—C12	−44.4 (4)
O1—C2—C3—O2	0.3 (6)	C18—O8—C12—C11	99.4 (4)
C1—C2—C3—O2	−178.7 (4)	C18—O8—C12—C13	−140.6 (4)
O1—C2—C3—C4	−179.1 (4)	O6—C11—C12—O8	−80.6 (4)
C1—C2—C3—C4	1.8 (6)	C10—C11—C12—O8	163.6 (3)
O2—C3—C4—C5	−178.4 (5)	O6—C11—C12—C13	162.6 (3)
C2—C3—C4—C5	1.0 (7)	C10—C11—C12—C13	46.8 (4)
C3—C4—C5—C6	−2.8 (8)	C9—O3—C13—C20	−169.9 (3)
C4—C5—C6—C1	1.8 (8)	C9—O3—C13—C12	66.9 (4)
C2—C1—C6—C5	0.9 (7)	O8—C12—C13—O3	−175.1 (3)
C8—C1—C6—C5	−175.7 (5)	C11—C12—C13—O3	−56.7 (4)
C10—N1—C8—C1	−175.9 (4)	O8—C12—C13—C20	64.9 (4)
C6—C1—C8—N1	175.8 (4)	C11—C12—C13—C20	−176.7 (3)
C2—C1—C8—N1	−0.8 (7)	C9—O4—C14—O5	−2.3 (8)
C13—O3—C9—O4	179.7 (3)	C9—O4—C14—C15	176.9 (4)
C13—O3—C9—C10	−65.7 (4)	C11—O6—C16—O7	1.7 (8)
C14—O4—C9—O3	−98.4 (4)	C11—O6—C16—C17	−177.7 (4)
C14—O4—C9—C10	144.0 (4)	C12—O8—C18—O9	3.0 (8)
C8—N1—C10—C11	−100.9 (4)	C12—O8—C18—C19	−177.5 (4)
C8—N1—C10—C9	137.6 (4)	C21—O10—C20—C13	−174.1 (4)
O3—C9—C10—N1	174.0 (3)	O3—C13—C20—O10	−66.5 (4)
O4—C9—C10—N1	−72.1 (4)	C12—C13—C20—O10	53.8 (5)
O3—C9—C10—C11	53.4 (4)	C20—O10—C21—O11	−0.8 (7)
O4—C9—C10—C11	167.3 (3)	C20—O10—C21—C22	178.7 (4)

### Hydrogen-bond geometry (Å, °)

**Table:**

*D*—H···*A*	*D*—H	H···*A*	*D*···*A*	*D*—H···*A*
O1—H1···N1	0.82	1.90	2.625 (4)	147
